# The “Bright Side” of Identity Fusion: Promoting Intentions to Engage in Intergroup Prosocial Behavior

**DOI:** 10.3390/bs16091701

**Published:** 2026-09-21

**Authors:** Jielin Shen, Sicen Shen, Yuchang Jin, Ping Zhang, Junxiu An, Peixuan Zheng

**Affiliations:** 1 College of Psychology, Sichuan Normal University, Chengdu 610066, China; shin0313@126.com (J.S.);; 2 College of Software Engineering, Chengdu University of Information Technology, Chengdu 610225, China; 3 Department of Kinesiology and Nutrition, University of Illinois Chicago, Chicago, IL 60612, USA

**Keywords:** identity fusion, group efficacy, empathy, intergroup prosocial behavior, Fusion–Secure Base Hypothesis

## Abstract

In increasingly diverse societies, intergroup prosocial behavior is crucial for improving intergroup relations and facilitating cross-group cooperation. Guided by the Fusion–Secure Base Hypothesis, the present research uses the North–South cultural divide in China as a meaningful intergroup boundary and examines whether identity fusion among Southern Chinese young adults is associated with intergroup prosocial behavior and through which psychological pathways. Study 1 employed a cross-sectional survey (*N* = 609) and showed that identity fusion was positively associated with intergroup prosocial behavior; group efficacy and empathy displayed parallel indirect effects linking identity fusion to intergroup prosocial behavior. Study 2 used a one-factor, three-level between-subjects experiment (*N* = 72) in which participants completed a Southern regional social self-reflection task, a personal self-reflection task, or a control task describing the laboratory setting. Results indicated that both activation groups reported higher intergroup prosocial intention than the control group, whereas the difference between the two activation groups was not significant. Exploratory mediation analyses suggested that group efficacy showed relatively more consistent preliminary evidence than empathy. Overall, the findings offer preliminary evidence that identity fusion is associated with intergroup prosocial behavior, while also suggesting that the underlying psychological pathways require further investigation.

## 1. Introduction

Modern societies are becoming increasingly diverse, making cooperation and mutual assistance across group boundaries increasingly important. Intergroup prosocial behavior refers to friendly or benevolent actions directed by ingroup members toward outgroup members, including helping, cooperation, and donations (Wright & Richard, 2009; Everett et al., 2015). Such group-level behavior not only helps reduce intergroup divisions, but may also provide a foundation for collaboration and improved relations between different groups. In research on the improvement of intergroup relations, intergroup contact theory and the Common Ingroup Identity Model (CIIM) represent two influential approaches, both of which have received empirical support. Intergroup contact theory holds that, under appropriate conditions, direct contact between members of different groups can reduce prejudice and improve intergroup attitudes (Pettigrew & Tropp, 2006; Van Assche et al., 2023). The CIIM, by contrast, suggests that when members of different groups recategorize themselves and others as belonging to a shared superordinate group, former outgroup members are incorporated into a more inclusive sense of “we,” thereby reducing prejudice and promoting more positive intergroup evaluations and behaviors (Gaertner & Dovidio, 2000; Lemay & Ryan, 2021; Cocco et al., 2024). Whereas these approaches emphasize behavioral contact and cognitive recategorization, less is known about whether strong bonds with the ingroup can shape prosocial intentions toward outgroup members. The present research addresses this question by focusing on identity fusion, a deep form of psychological connectedness in which individuals experience a high degree of oneness with their ingroup and a close integration of the personal self and the social self.

Identity fusion is defined as a strong visceral feeling of oneness between the individual and the group (Swann et al., 2009, 2012). In Comprehensive Identity Fusion Theory (CIFT), identity fusion is conceptualized as a synergistic union between the personal self—that is, the aspects of the self that are unique to the individual—and the fusion target. A defining feature of this union is the presence of porous and permeable borders between the personal self and the fusion target (Swann et al., 2024). These porous borders do not imply that the personal self is absorbed by the group identity, nor do they simply indicate a blurring of boundaries between the two. Rather, they generate identity synergy, through which individuals direct their personal agency toward behaviors that support the fusion target (Swann et al., 2024). Gómez et al. (2025a) adopt a similar understanding, describing fusion as a synergistic union with a group, individual, or cause, which can further promote behavioral support for the fusion target.

According to Swann et al.’s (2024) summary of the classic social identity perspective, traditional social identity theory and self-categorization theory are often considered to contain two assumptions that contrast with identity fusion theory. The first is the depersonalization hypothesis, which suggests that when group identity becomes salient, the social self overshadows or weakens the personal self. The second is the functional antagonism hypothesis, which holds that the activation of social identity and personal identity is competitive and incompatible. Identity fusion theory, by contrast, argues that when highly fused individuals engage with the group, their personal identity remains active and operates in synergy with, rather than being suppressed by their social identity (Swann et al., 2024). In addition, identity fusion emphasizes the distinctive role of relational ties between individuals and ingroup members: weakening relational ties with ingroup members reduces identity fusion, but does not reduce overall group identification to the same extent (Gómez et al., 2019). Thus, compared with traditional group identification, identity fusion places greater emphasis on the joint role of the personal self, the social self, and relational ties in motivating pro-group behavior, rather than viewing individual identity as being unilaterally subsumed under group identity. Thus, identity fusion theory provides an appropriate theoretical framework for understanding the question addressed in the present study: how strong ingroup bonds may, under certain conditions, extend outward into cross-group interaction and intergroup prosocial behavior. It not only builds on the traditional line of research explaining costly pro-group behavior, but also, by emphasizing that the personal self is not absorbed by group identity, offers explanatory space for understanding how individuals can maintain strong ingroup bonds while remaining open and trusting toward outgroups.

Identity fusion theory was originally developed primarily in the context of explaining extreme pro-group behavior, focusing on why individuals are willing to fight or die for their group (Swann et al., 2009, 2012; Gómez et al., 2011). Early studies showed that highly fused individuals were more willing to engage in costly behavior on behalf of the group, and that this tendency became more pronounced when either their personal identity or social identity was activated (Swann et al., 2009). Notably, the “fight and die for the group” scale commonly used in identity fusion research typically presents its items in contexts involving group protection or responses to group threat (Swann et al., 2009, 2024; Gómez et al., 2011), suggesting that threat contexts constitute an important background for understanding the relationship between identity fusion and extreme pro-group behavior. As research has progressed, however, scholars have begun to examine whether identity fusion may also produce positive intergroup consequences under theoretically low-conflict conditions. The Fusion–Secure Base Hypothesis proposed by Klein and Bastian (2023) provides a theoretical explanation for this possibility. According to this hypothesis, in the absence of outgroup threat, the fused group may function as a “secure base,” allowing members to feel safe and supported and thereby making them more likely to engage with outgroups.

By contrast, when an outgroup is perceived as threatening, fusion may elicit defensive or even aggressive pro-group responses (Klein & Bastian, 2023). Consistent with this view, Vázquez et al. (2023) found that, in familiar and non-threatening outgroup contexts, highly fused individuals expressed more positive affect toward outgroup members than did less fused individuals, whereas negative intergroup contact led highly fused individuals to derogate outgroup members. Klein et al. (2024) further found that identity fusion was consistently and positively associated with general trust, outgroup trust, and social exploration. Their internal meta-analysis further showed that, compared with several measures of group identification, identity fusion was more strongly associated with trust and social exploration. More recently, Klein et al. (2025) found that when an outgroup was perceived positively, or when intergroup cooperation was seen as beneficial to the ingroup, identity fusion was more likely to positively predict outgroup trust and willingness to cooperate.

Together, these studies suggest that identity fusion is more likely to lead to extreme pro-group responses in threatening contexts; however, in familiar and non-threatening outgroup contexts, it may also be associated with outgroup trust, social exploration, and positive affect, thereby facilitating individuals’ engagement in cross-group interaction and prosocial behavior. The present study extends this question to the context of North–South regional identity in China. Chinese North–South regional identity is grounded in certain historical, geographical, and cultural-psychological differences. Previous research has shown that regional psychological differences within China are associated with rice- and wheat-farming traditions (Talhelm et al., 2014). However, unlike high-threat intergroup contexts such as war or prolonged violent conflict, Northern and Southern regional groups are both internal regional groups within China. Under a shared national identity, they retain certain regional and cultural boundaries, but they do not constitute a typical hostile or threatening intergroup relationship. Precisely because it combines a shared superordinate identity, regional cultural boundaries, and low levels of conflict, the Chinese North–South regional identity context provides a valuable setting for examining intergroup prosocial behavior in a context that is “familiar yet culturally bounded.” At the same time, whether helpers’ willingness to provide assistance can be translated into positive intergroup outcomes also depends on how recipients interpret help from outgroup members. Previous research has shown that help offered by outgroup members is often attributed to weaker prosocial motives and is less likely to be accepted by recipients, especially when prejudice is high or when the outgroup is described negatively. Tolerant ingroup norms, however, can significantly reduce or even eliminate recipients’ suspicion toward help from outgroup members (Borinca et al., 2021, 2022). In the Chinese North–South context, as noted above, the level of conflict between Northern and Southern groups is relatively low, and the two groups are relatively familiar with one another. These contextual features are similar to the tolerant-norm and low-prejudice conditions examined by Borinca and colleagues, suggesting that, in the context of the present study, fused individuals’ willingness to help may have relatively favorable conditions for leading to positive intergroup outcomes. Accordingly, the present study proposes the following hypothesis:

**H1.**  *In the relatively low-conflict and non-hostile context of North–South regional relations in China, stronger identity fusion with the Southern regional ingroup among Southern Chinese young adults will be positively associated with stronger intergroup prosocial intentions toward Northerners*.

If identity fusion can positively predict intergroup prosocial behavior, what are the mechanisms underlying this relationship? The influence of identity fusion on group-related behavior may involve multiple psychological processes, and Gómez et al. (2025b) provided a systematic review of the mechanisms underlying identity fusion effects and the processes through which these effects translate into behavioral outcomes. Building on recent developments in this field, the present study focuses on two pathways: group efficacy and empathy. This focus is primarily based on their theoretical complementarity. Group efficacy corresponds to a competence–agency pathway, reflecting how highly fused individuals may transform their sense of personal agency into beliefs about the group’s collective capacity. Empathy, by contrast, corresponds to an affect–care pathway, representing how the relational connectedness embedded in fusion may provide an emotional basis for empathic concern. These two pathways explain why identity fusion may promote intergroup prosocial behavior from two complementary perspectives: “we are capable of taking helpful action” and “others’ needs can elicit affective concern.”

Group efficacy refers to members’ beliefs in the group’s shared capability, that is, the belief that the group can organize and execute the actions required to attain specific goals (Bandura, 1997; Stajkovic et al., 2009). Research has shown that group efficacy is closely related to group performance. When group members believe that the group is capable of handling a given task, this belief influences whether the group initiates action, how much effort it invests, and how long such effort is sustained (Stajkovic et al., 2009). In research on collective action, group efficacy is regarded as an important pathway influencing collective action tendencies, reflecting the extent to which individuals believe that collective action can effectively address the group’s circumstances (Van Zomeren et al., 2004). Unlike general group identification, identity fusion emphasizes the strong connection between the personal self and the social self. Highly fused individuals do not merely regard themselves as members of the group; rather, they are more likely to link their personal agency to the group, to feel that their own actions can become part of collective action, and to believe that they can have an impact on the group. Gómez et al. (2011) showed that fused individuals’ sense of connectedness with the group and reciprocal strength may lead them to tether their personal agency to the group. They further found that agency and invulnerability mediated the relationship between identity fusion and extreme pro-group behavior. Besta et al. (2018) found associations among identity fusion, group efficacy, and collective action tendencies, and showed that group efficacy could explain the relationship between identity fusion and collective action tendencies. These findings suggest that identity fusion may make individuals more likely to transform the feeling that “I can act” into the group-level belief that “we can act.” In the North–South intergroup context examined in the present study, group efficacy is understood as a capacity belief that supports constructive cross-group interaction. Although real cultural boundaries exist between Northern and Southern groups in China, these groups are not highly antagonistic and share a broader national and cultural community. In such a low-conflict intergroup context, higher group efficacy may lead individuals to believe that their ingroup has sufficient resources, confidence, and capacity for action to engage in cross-group helping, thereby reducing concerns about the costs of helping or the uncertainty involved in intergroup interaction.

**H2.**  *Accordingly, the present study proposes that group efficacy will mediate the association between identity fusion and intergroup prosocial intentions*.

In addition to the competence–agency pathway represented by group efficacy, the present study further examines empathy as an affect–care pathway. According to the empathy–altruism hypothesis, empathic concern is an emotional response directed toward another person’s welfare. When individuals perceive that others are in need of help and experience empathic concern, they are more likely to develop a helping motivation aimed at improving the other person’s situation (Batson et al., 2015). In intergroup relations research, empathy has also been regarded as an important psychological process for improving intergroup attitudes and promoting help toward outgroup members. Empathy for a specific member of a stigmatized group can, under certain conditions, improve attitudes toward the group as a whole (Batson et al., 1997). Perspective-taking, empathic concern, and cues to a shared identity can also help reduce intergroup prejudice and promote positive responses toward outgroup members (Dovidio et al., 2010). The relationship between identity fusion and empathy can be understood in terms of the relational connectedness embedded in fusion. In their discriminant validity tests, Gómez et al. (2011) found that identity fusion was only weakly related to general trait empathy, indicating that the two constructs are conceptually independent. However, a weak direct association does not rule out the possibility of indirect and context-dependent links between them. National identity fusion can evoke empathic concern through relational ties, and empathic concern, in turn, positively predicts self-sacrificial behavior on behalf of the nation (Landabur et al., 2022). Although that study focused on pro-ingroup sacrifice, it suggests that the relational connectedness involved in identity fusion may provide an emotional basis for empathic concern.

In the North–South regional context, which is characterized by low conflict, low threat, and a shared superordinate identity, the relational connectedness and sense of responsibility for action elicited by identity fusion may be further translated, through general empathic tendencies, into willingness to help or donate to outgroup members. Research has shown that when the outgroup is familiar and non-threatening, highly fused individuals may express more positive affective evaluations of outgroup members (Vázquez et al., 2023). This suggests that identity fusion does not necessarily lead to exclusionary or defensive responses; rather, under certain conditions, it may support positive intergroup reactions. The empathy measured in the present study primarily reflects individuals’ general empathic tendency, rather than specific intergroup empathy directed toward Northerners. Therefore, this study conceptualizes empathy as a relatively general affect–care mechanism: the relational connectedness and sense of responsibility for action elicited by identity fusion may be more readily translated, through general empathic tendencies, into prosocial behavior toward outgroup members.

**H3.**  *Therefore, the present study proposes that empathy will mediate the association between identity fusion and intergroup prosocial intentions*.

Based on the logic of identity synergy in identity fusion theory discussed above, identity fusion does not mean that the personal self is replaced by group identity. On the contrary, highly fused individuals retain a strong personal self and connect it with the social self. Swann et al. (2009) proposed that, in a state of identity fusion, personal identity and social identity are functionally equivalent: activating either the personal self or the social self may motivate group-related behavior among fused individuals, and the two identities may mutually reinforce one another. Their series of studies showed that highly fused individuals reported a stronger willingness to fight and die for the group when either their personal identity or social identity was activated. In particular, Study 3 provided direct evidence for the functional equivalence of the two identities among fused individuals by comparing personal-identity activation, social-identity activation, and control conditions. Gómez et al. (2011) further provided empirical support for the role of the personal self in identity fusion. Their findings showed that activations or challenges related to the personal self could increase highly fused individuals’ endorsement of extreme pro-group behavior. At the same time, personal agency and invulnerability explained the relationship between identity fusion and extreme pro-group behavior. These findings suggest that, for highly fused individuals, the personal self is not an individual-level component irrelevant to group action; rather, it may become an important factor shaping group-related action. However, the studies discussed above mainly examined pro-ingroup behavior, such as fighting or dying for the ingroup, rather than prosocial behavior directed toward outgroup members.

Therefore, in the present study, we further examine whether the logic of identity synergy in identity fusion can be extended to constructive cross-group helping behavior in the North–South Chinese intergroup context, which is characterized by real cultural boundaries, relatively low levels of conflict, and a shared superordinate identity. In other words, when the personal self or the social self is activated, the group-related action motivation of highly fused individuals may be expressed not only as ingroup protection, but also as helping or donating to outgroup members.

**H4.**  *Accordingly, we hypothesize that participants in both the personal self and social self-activation conditions will report stronger intergroup prosocial tendencies than those in the control condition.*

It should be noted that there is still a lack of direct theoretical and empirical evidence regarding the relative effects of personal self-priming and social self-priming on intergroup prosocial behavior. The functional equivalence proposition proposed by Swann et al. (2009) mainly indicates that, for highly fused individuals, both personal identity and social identity may motivate willingness to act on behalf of the group. Existing priming studies have also focused primarily on pro-ingroup behavior and have rarely examined whether the two types of self-priming differ in their effects in outgroup helping contexts. Therefore, the present study examines this issue as an exploratory research question: Do personal self and social self-activation differ in their effects on intergroup prosocial tendencies? (RQ1)

**Figure 1:**
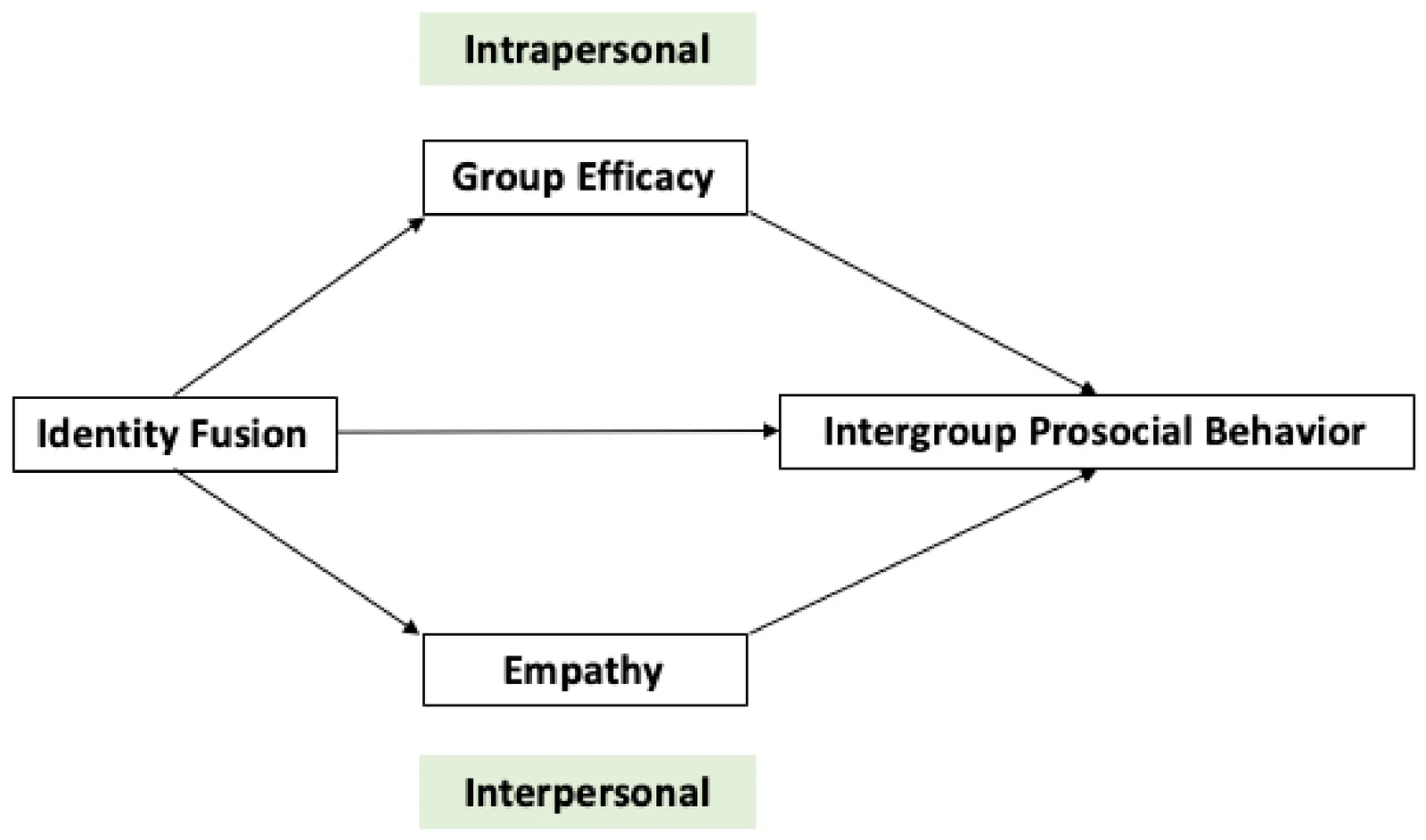
Conceptual parallel mediation model.

In summary, the present study focuses on Southern Chinese young adults aged 18 to 35 and develops a theoretical model of how identity fusion influences intergroup prosocial behavior in the North–South Chinese intergroup context, which involves real cultural boundaries, low levels of conflict, and a shared superordinate identity (see Figure 1). This model aims to extend identity fusion research from the pro-ingroup behaviors emphasized in the previous literature to the context of outgroup helping, and to explain its positive intergroup consequences through a competence–agency pathway and an affect–care pathway. Specifically, Study 1 examines the mediating roles of group efficacy and empathy in the relationship between identity fusion and intergroup prosocial behavior. Study 2 uses personal self and social self-priming to test whether the logic of identity synergy in identity fusion can be extended to prosocial behavior in a low-conflict intergroup context. In doing so, the present study provides new empirical evidence for understanding the positive intergroup consequences of identity fusion and the mechanisms through which they occur.

Study 1: Employed an exploratory cross-sectional design to examine associations among identity fusion, group efficacy, empathy, and intergroup prosocial behavior.

## 2. Study 1: Method

### 2.1. Participants and Design

Using convenience sampling, we recruited participants aged 18–35 from key regions of Southern China (Sichuan Province, Guangdong Province, and Chongqing Municipality). Participants were recruited through both online and offline recruitment channels. Regardless of the recruitment channel, all participants completed the same online questionnaire. Participants received small gifts as compensation. Using convenience sampling, we recruited 647 participants from Southern China. To precisely identify the Southern group, we included the screening item “Do you self-identify as a Southerner?” Participants who answered “No” were excluded. All items were adapted to refer to Southerners/the Southern group, and participants received standardized instructions before responding. After excluding outliers and invalid responses, 609 valid questionnaires were retained (valid response rate = 94.13%).

### 2.2. Measures

#### 2.2.1. Identity Fusion

Identity fusion was measured using the Identity Fusion Scale (Gómez et al., 2011). The scale includes 7 items (1 = strongly disagree, 7 = strongly agree). A sample item is: “I feel that I am fused with the Southern region to which I belong.” All items are positively worded. Higher total scores indicate greater levels of identity fusion. In this study, Cronbach’s *α* = 0.92.

#### 2.2.2. Group Efficacy

Group efficacy was measured using the Group Efficacy Scale (Shi & Cui, 2014), adapted from Van Zomeren et al. (2010) and revised to fit the Chinese context. The scale includes 4 items (1 = strongly disagree, 7 = strongly agree). A sample item is: “The group I belong to (the Southern group) is fully capable of accomplishing various activities.” All items are positively worded. Higher total scores indicate greater group efficacy. In this study, Cronbach’s *α* = 0.93.

#### 2.2.3. Empathy

Empathy was measured using the Interpersonal Reactivity Index (IRI) (Davis, 1980), revised by Zhang (1987) to fit the Chinese context. Items are rated from 0 (does not describe me well) to 4 (describes me very well). The scale includes 22 items. A sample item is: “Before making a decision, I try to look at everybody’s side of a disagreement.” This scale consists of four factors: Perspective Taking, Empathic Concern, Fantasy, and Personal Distress. Items 2, 5, 10, 11, and 14 are reverse-scored. After adjusting for reverse-scored items, higher total scores indicate higher levels of empathy. In this study, Cronbach’s *α* = 0.76.

#### 2.2.4. Intergroup Prosocial Intention

Intergroup prosocial intention was measured using the Intergroup Prosocial Behavior Scale (DeWall et al., 2008), revised by Chang and Xie (2020). As the measure consisted of hypothetical helping scenarios, we refer to the assessed outcome as intergroup prosocial intention rather than actual intergroup prosocial behavior. Before completing the scale, participants were instructed that, as Southern Chinese, all helping targets described in the following scenarios were Northern Chinese residents, thereby explicitly establishing the intended regional intergroup boundary. The measure consisted of four hypothetical helping scenarios involving different forms of assistance toward Northern Chinese outgroup members: helping an elderly woman from a poor Northern area, supporting economically disadvantaged students from Northern China, donating to rural libraries serving children in Northern China, and donating to post-earthquake reconstruction for residents in Qinghai Province in Northern China. Participants rated their willingness to provide help on a 7-point Likert scale ranging from 1 (not willing at all) to 7 (completely willing). A sample item is: “Would you be willing to donate to a rural library serving children in Northern China?” All items were positively worded, with higher scores indicating greater intergroup prosocial behavior. In the present study, Cronbach’s α was 0.92.

A Harman single-factor test yielded eight factors with eigenvalues greater than 1; the first factor accounted for 25.78% of the variance, suggesting no severe risk of common method bias.

### 2.3. Analytic Strategy

We conducted correlation and regression analyses among identity fusion, intergroup prosocial intention, group efficacy, and empathy. We then tested the mediating roles of group efficacy and empathy between identity fusion and intergroup prosocial intention while controlling for gender, age, and birthplace.

### 2.4. Results

Because Study 1 was cross-sectional, the mediation model was interpreted as a statistical indirect-effect model rather than as evidence of causal mediation. As shown in Table 1, identity fusion was positively correlated with intergroup prosocial intention, *r* = 0.40, *p* < 0.001, group efficacy, *r* = 0.75, *p* < 0.001, and empathy, *r* = 0.13, *p* < 0.001. Group efficacy was also positively correlated with intergroup prosocial behavior, *r* = 0.37, *p* < 0.001, and empathy, *r* = 0.11, *p* < 0.001. In addition, empathy was positively correlated with intergroup prosocial intention, *r* = 0.38, *p* < 0.001. These correlations provided preliminary support for testing the mediation analyses.

We tested a parallel indirect-effect model with identity fusion as the predictor, intergroup prosocial intention as the outcome variable, and group efficacy and empathy as parallel intervening variables. Because all variables in Study 1 were measured at the same time, this model was interpreted as a test of cross-sectional indirect associations. First, identity fusion significantly and positively predicted intergroup prosocial intention, *β* = 0.40, *t* = 10.85, *p* < 0.001. When identity fusion, group efficacy, and empathy were entered simultaneously, identity fusion also significantly predicted group efficacy, *β* = 0.75, *t* = 28.17, *p* < 0.001, and empathy, *β* = 0.13, *t* = 3.30, *p* < 0.001; group efficacy positively predicted intergroup prosocial intention, *β* = 0.16, *t* = 2.84, *p* < 0.01, as did empathy, *β* = 0.33, *t* = 9.49, *p* < 0.001. The direct path from identity fusion to intergroup prosocial intention remained significant, *β* = 0.25, *t* = 4.71, *p* < 0.001.

**Figure 2:**
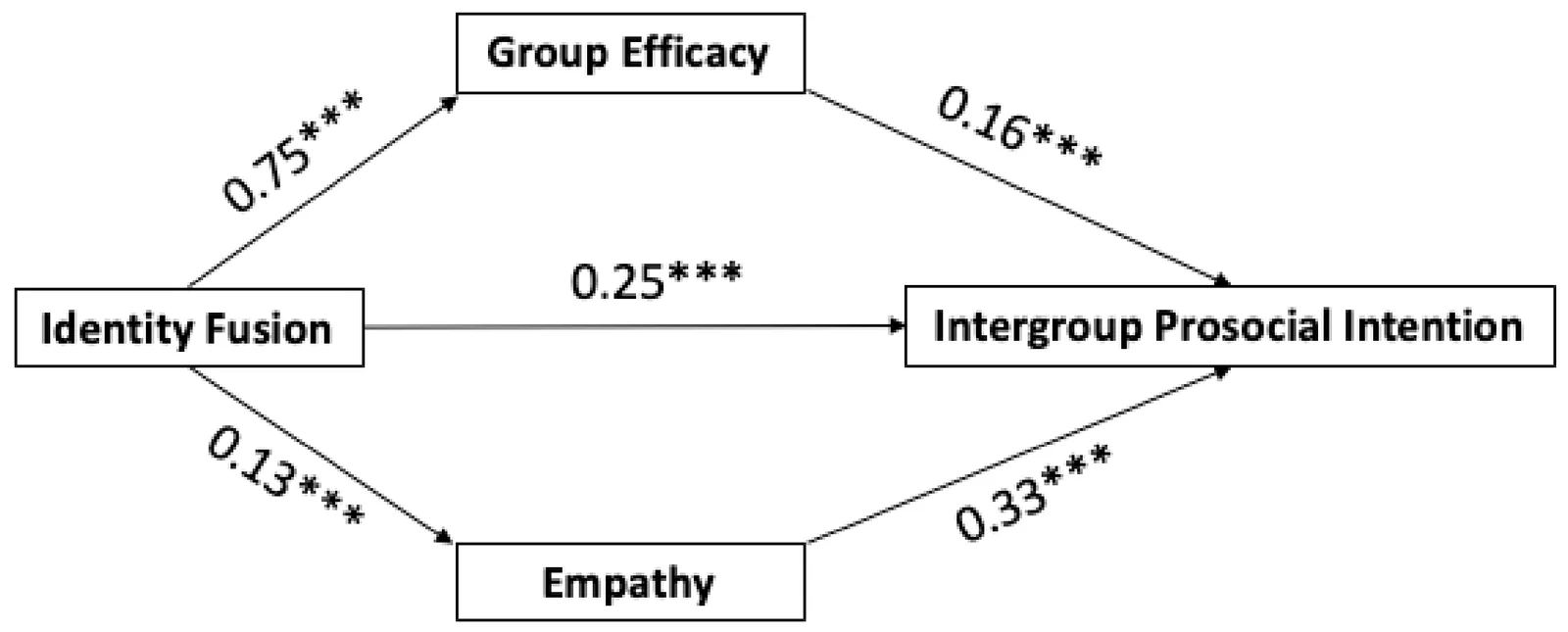
Statistical Mediation Model Path Diagram. Note. Standardized regression coefficients (β) are shown on the paths. *** *p* < 0.001.

Bootstrapping analyses further showed that the indirect effect through group efficacy was significant, *effect* = 0.06, *BootSE* = 0.02, *95% CI* [0.02, 0.10]. The indirect effect through empathy was also significant, *effect* = 0.02, *BootSE* = 0.01, *95% CI* [0.01, 0.04]. Thus, identity fusion was indirectly associated with intergroup prosocial intention through both group efficacy and empathy. Standardized coefficients are reported for the regression paths, whereas unstandardized estimates are reported for the bootstrapped direct, indirect, and total effects. These findings suggest that identity fusion was statistically associated with intergroup prosocial intention both directly and indirectly through group efficacy and empathy. Please see Table 2 and Figure 2.

Given the strong correlation between identity fusion and group efficacy, we conducted additional analyses to examine whether the two constructs were empirically distinguishable and whether multicollinearity was problematic. First, a confirmatory factor analysis compared a two-factor model, in which identity fusion and group efficacy items loaded onto separate latent factors, with a one-factor model, in which all items loaded onto a single latent factor. The two-factor model showed better fit, χ^2^(43) = 434.14, CFI = 0.927, TLI = 0.906, RMSEA = 0.122, SRMR = 0.047, AIC = 20,021.28, BIC = 20,171.28, than the one-factor model, χ^2^(44) = 963.69, CFI = 0.828, TLI = 0.785, RMSEA = 0.185, SRMR = 0.070, AIC = 20,548.83, BIC = 20,694.42. Standardized factor loadings were acceptable to high for identity fusion (0.645–0.833) and group efficacy (0.853–0.913). Although the latent correlation between identity fusion and group efficacy was high, *r* = 0.807, 95% CI [0.759, 0.855], the superior fit of the two-factor model supports their empirical distinctiveness.

Second, multicollinearity diagnostics were conducted for the regression model predicting intergroup prosocial intentions from identity fusion, group efficacy, empathy, gender, age, and birthplace. The VIF values ranged from 1.019 to 2.354, and tolerance values ranged from 0.425 to 0.981. The VIF values for identity fusion and group efficacy were 2.315 and 2.354, respectively, suggesting that severe multicollinearity was unlikely.

### 2.5. Study 1 Discussion

Study 1 provided initial support for H1, H2, and H3. Consistent with H1, identity fusion was positively associated with intergroup prosocial intention among Southern Chinese young adults. Consistent with H2 and H3, the parallel indirect-effect model showed that identity fusion was indirectly associated with intergroup prosocial intention through both group efficacy and empathy. However, Study 1 also had several limitations. First, because all variables were measured at the same time using self-report questionnaires, the results should be interpreted as correlational rather than causal. Second, although the helping scenarios explicitly identified the recipients as Northern Chinese outgroup members, all scenarios involved recipients experiencing hardship, which may have activated general humanitarian or moral concerns. It is important to interpret these findings cautiously. Although group efficacy and empathy showed significant indirect associations between identity fusion and intergroup prosocial intention, the cross-sectional design of Study 1 prevents conclusions about temporal order or causal mechanisms. Therefore, these results should be understood as preliminary evidence of statistically indirect associations rather than evidence that group efficacy or empathy causally explains the effect of identity fusion on prosocial intention. Building on these initial correlational findings, Study 2 sought to provide stronger inferential evidence by experimentally activating self-representations related to identity fusion.

Study 2: Because the survey design in Study 1 can only establish associations and cannot support causal inference, Study 2 used an experimental design. By activating self-representations related to identity fusion (personal self vs. social self), we simulated the psychological basis of a fused state and examined whether activating self-representations related to identity fusion influenced intergroup prosocial intention. Study 2 used a one-factor, three-level between-subjects design, controlling for age, gender, and birthplace.

## 3. Study 2: Method

### 3.1. Participants

Approximately four months after Study 1, we invited a subset of eligible participants from the Study 1 participant pool to take part in Study 2. To reduce potential baseline differences in intergroup prosocial intention and to minimize possible ceiling effects, participants were selected based on their Study 1 intergroup prosocial intention scores: only those whose scores were below the sample mean and did not differ significantly at baseline were invited. All participants were from Sichuan—one of the Southern provinces—and had long-term residence experience in Sichuan. Power analysis using G*Power 3.1 indicated a minimum sample size of 66 for an F-test (*α* = 0.05, effect size f = 0.40 based on Cohen, 1988, power = 0.80). Eighty participants took part. Participants reported no major life events or substantial mood fluctuations recently. After excluding eight invalid cases, the final sample comprised 72 participants (35 men, 37 women; *M*_age = 23.01). Each participant received 20 RMB after the experiment.

### 3.2. Procedure

The experimental manipulation was adapted from the identity-activation procedure used by Swann et al. (2014, Experiment 6). In their study, participants were assigned to a personal self-activation condition, a social self-activation condition, or a control condition. Participants in the personal self condition were asked to think about their personal characteristics and preferences; those in the social self condition were asked to think about themselves as members of their national group; and those in the control condition were asked to objectively describe the place where they were completing the questionnaire. We adapted this procedure to the Southern Chinese regional context by changing the group reference from national identity to Southern regional identity and by pairing the reflection tasks with brief video materials relevant to each condition.

Participants were randomly assigned to one of three conditions: social self-activation, personal self-activation, or control. Before the experimental manipulation, all participants completed a pre-manipulation phase. They first provided basic demographic and screening information, including age, gender, registered residence, whether they had participated in similar experiments, and whether their reading comprehension was normal. They then completed a pre-test measure of identity fusion using the verbal identity fusion scale. Next, participants completed a 5 min relaxation exercise to reduce the potential influence of their immediate pre-experimental state. This pre-manipulation phase lasted approximately 10 min.

In the social self-activation condition, participants first watched the promotional video *Broad and Narrow Sichuan* (3 min 47 s), provided by the Sichuan Provincial Department of Culture and Tourism. They were then asked to reflect on their social self as members of their hometown in Southern China. Specifically, they wrote about iconic places and buildings in their Southern hometown, well-known local figures, local food, Southern cultural customs, words that described their hometown, and what made them feel proud as a member of their hometown of Southern China. They wrote down their reflections.

In the personal self-activation condition, participants first watched the bilingual-subtitled YouTube video *How to Know Yourself* (edited to 3 min 45 s). They were then asked to reflect on their personal self, including their hobbies, strengths, preferences (e.g., food, clothing), areas of expertise, and key personality traits. They wrote down their reflections. The written reflections were included as part of the self-activation procedure. The primary manipulation check was the post-manipulation identity fusion score, compared with the pre-manipulation baseline measure.

**Figure 3:**
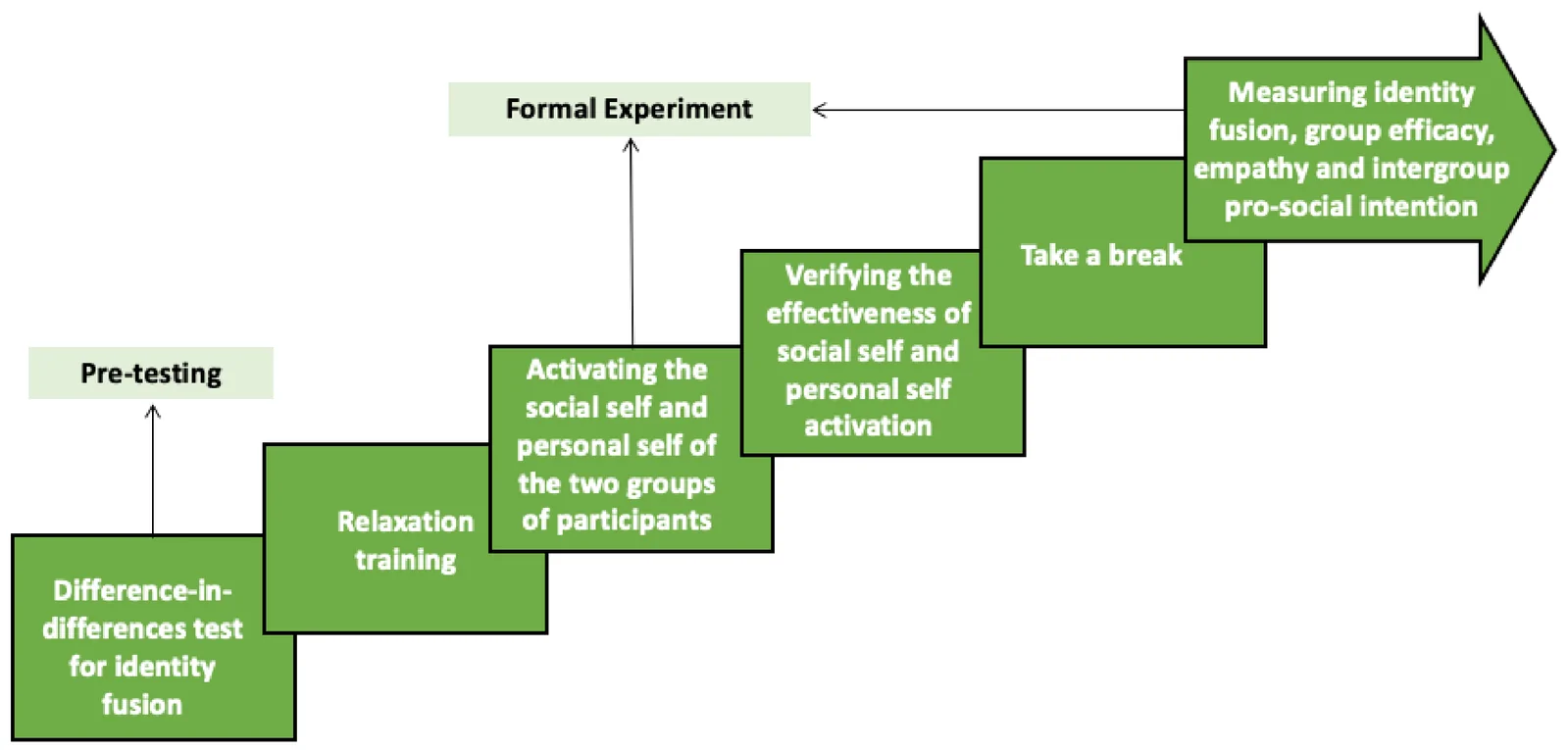
Flowchart of the experiment.

In the control condition, participants did not complete a self-related reflection task. Instead, they were asked to observe and describe the physical features of the laboratory setting. After the manipulation, all participants completed the post-manipulation Identity Fusion Scale, Group Efficacy Scale, Intergroup Prosocial Behavior Scale, and Interpersonal Reactivity Index (as in Study 1). The overall procedure is shown in Figure 3.

### 3.3. Measures

The measures used in Study 2 were the same as those used in Study 1, with wording adapted to the Southern Chinese regional context.

#### 3.3.1. Identity Fusion

Identity fusion was measured using the 7-item verbal Identity Fusion Scale (Gómez et al., 2011) as in Study 1. Participants completed this scale twice: once before the experimental manipulation to assess baseline identity fusion, and once after the manipulation as a manipulation check. Items referred to participants’ fusion with their Southern regional group. Higher scores indicated stronger identity fusion.

#### 3.3.2. Group Efficacy

Group efficacy was measured using the 4-item Group Efficacy Scale adapted from Van Zomeren et al. (2010) and revised by Shi and Cui (2014). Items assessed participants’ perceived efficacy of their Southern regional group. Higher scores indicated greater group efficacy.

#### 3.3.3. Empathy

Empathy was assessed using the Chinese version of the Interpersonal Reactivity Index (IRI; Davis, 1980; Zhang, 1987). Higher scores indicated higher dispositional empathy.

#### 3.3.4. Intergroup Prosocial Intention

Intergroup prosocial intention was measured using the Intergroup Prosocial Behavior Scale (DeWall et al., 2008), revised by Chang and Xie (2020). As in Study 1, participants were instructed that the helping targets were Northern Chinese residents, thereby establishing the intended regional intergroup boundary. Participants rated their willingness to help across hypothetical helping scenarios, with higher scores indicating stronger intergroup prosocial intention.

### 3.4. Results

#### 3.4.1. Manipulation Check: Pre- and Post-Manipulation Identity Fusion

To examine whether the three experimental groups differed in identity fusion before the manipulation, we first conducted a one-way ANOVA on pre-manipulation identity fusion scores. The results showed no significant difference among the social self-activation group (*M* = 35.50, *SD* = 4.16), personal self-activation group (*M* = 34.21, *SD* = 3.96), and control group (*M* = 35.38, *SD* = 4.12), *F*(2, 69) = 0.73, *p* > 0.05; see Table 3. This suggests that the three groups were comparable in baseline identity fusion.

We then conducted a one-way ANOVA on post-manipulation identity fusion scores measured after the manipulation. The results showed a significant difference among the three groups, *F*(2, 69) = 5.94, *p* < 0.01; see Table 4. Post hoc comparisons indicated that the social self-activation group (*M* = 38.21, *SD* = 4.23) reported higher identity fusion than the personal self-activation group (*M* = 35.67, *SD* = 3.60), whereas the personal self-activation group did not differ significantly from the control group (*M* = 34.42, *SD* = 3.80). These results suggest that social self-activation was associated with higher post-manipulation identity fusion, whereas the effect of personal self-activation on identity fusion was less evident.

#### 3.4.2. Effects of Experimental Condition on Intergroup Prosocial Intention

We conducted a one-way ANOVA to examine whether intergroup prosocial intention differed across the three experimental conditions. The results showed a significant effect of experimental condition, *F*(2, 69) = 30.67, *p* < 0.001, *η*^2^ = 0.47.

**Figure 4:**
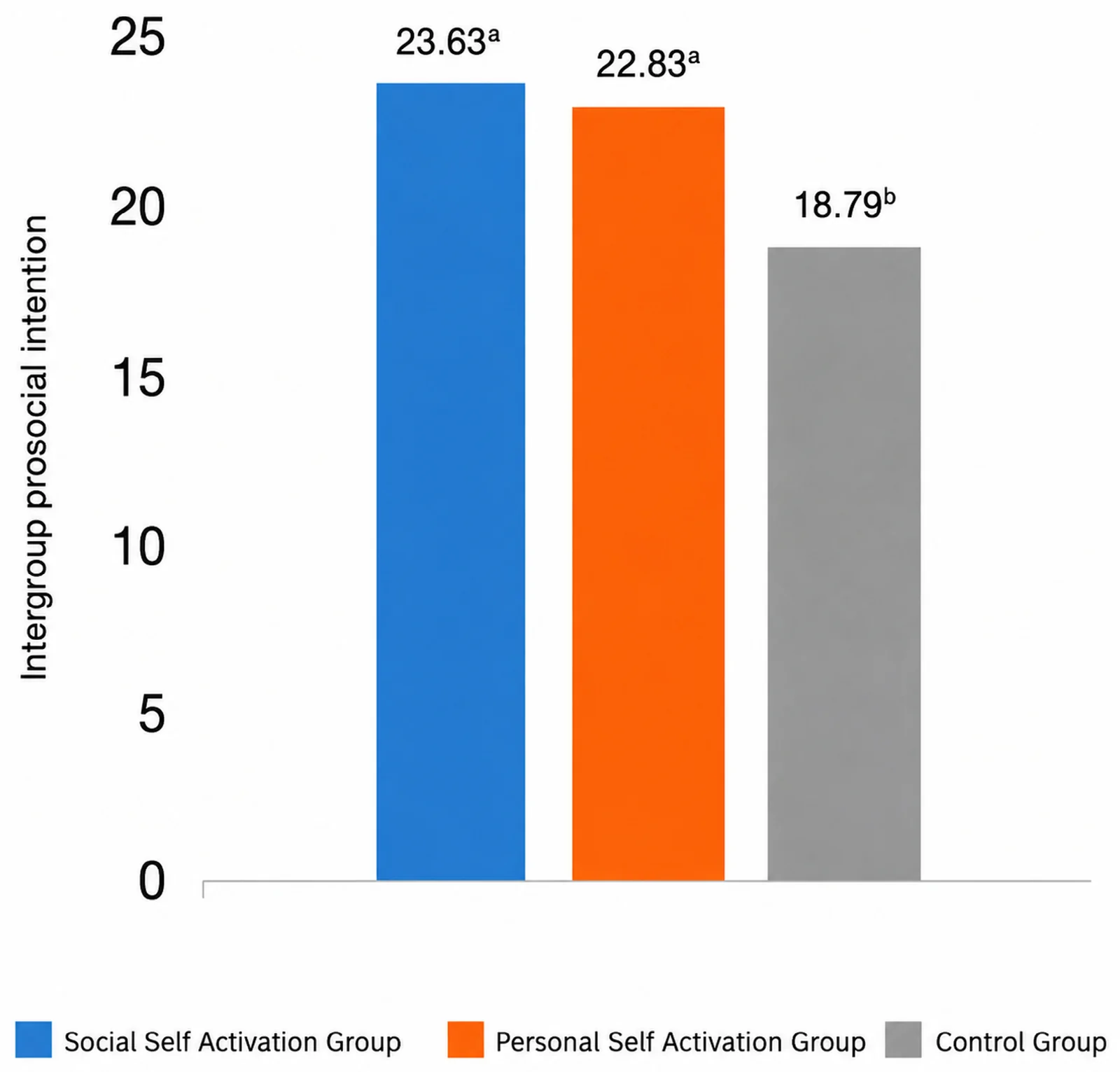
Mean intergroup prosocial intention across the three experimental conditions. Note: Different lowercase superscript letters indicate statistically significant differences between conditions at *p* < 0.001; conditions sharing the same letter did not differ significantly.

Tukey post hoc comparisons indicated that participants in the social self-activation condition reported significantly higher intergroup prosocial intention than those in the control condition, mean difference = 4.83, *95% CI* [3.25, 6.42], *p* < 0.001. Participants in the personal self-activation condition also reported significantly higher intergroup prosocial intention than those in the control condition, mean difference = 4.04, *95% CI* [2.46, 5.63], *p* < 0.001. The difference between the social self and personal self-activation conditions was not statistically significant, mean difference = 0.79, *95% CI* [−0.79, 2.38], *p* = 0.460, as shown in Figure 4. Given the sample size, this non-significant difference should not be interpreted as evidence that the two active conditions had equivalent effects.

#### 3.4.3. Mediation Analysis

Given the small sample size within each experimental condition, the mediation analyses should be interpreted as exploratory. These analyses were conducted to examine whether the indirect-association pattern observed in Study 1 appeared descriptively similar after the activation tasks, rather than to provide a definitive test of mediation within each condition.

**Figure 5:**
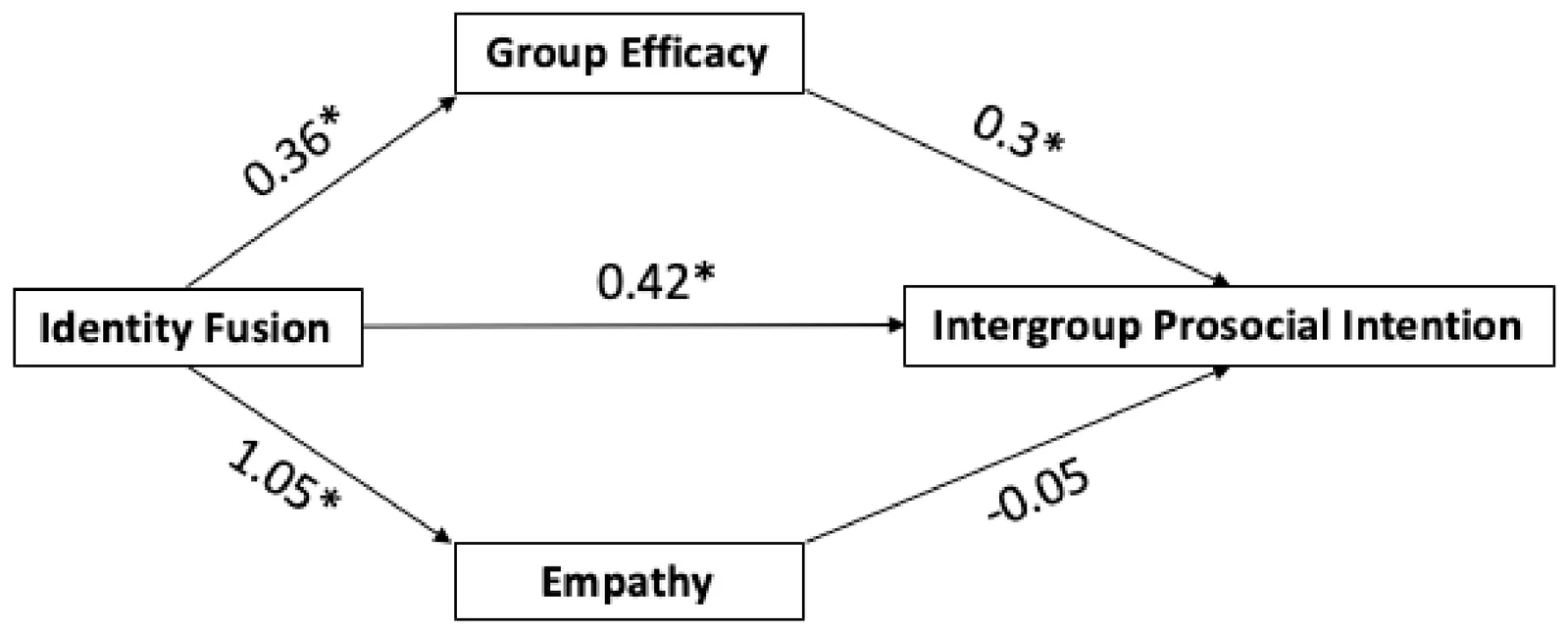
Statistical Mediation Model Path for Social Self-Activation Group. Note: * *p* < 0.05.

**Figure 6:**
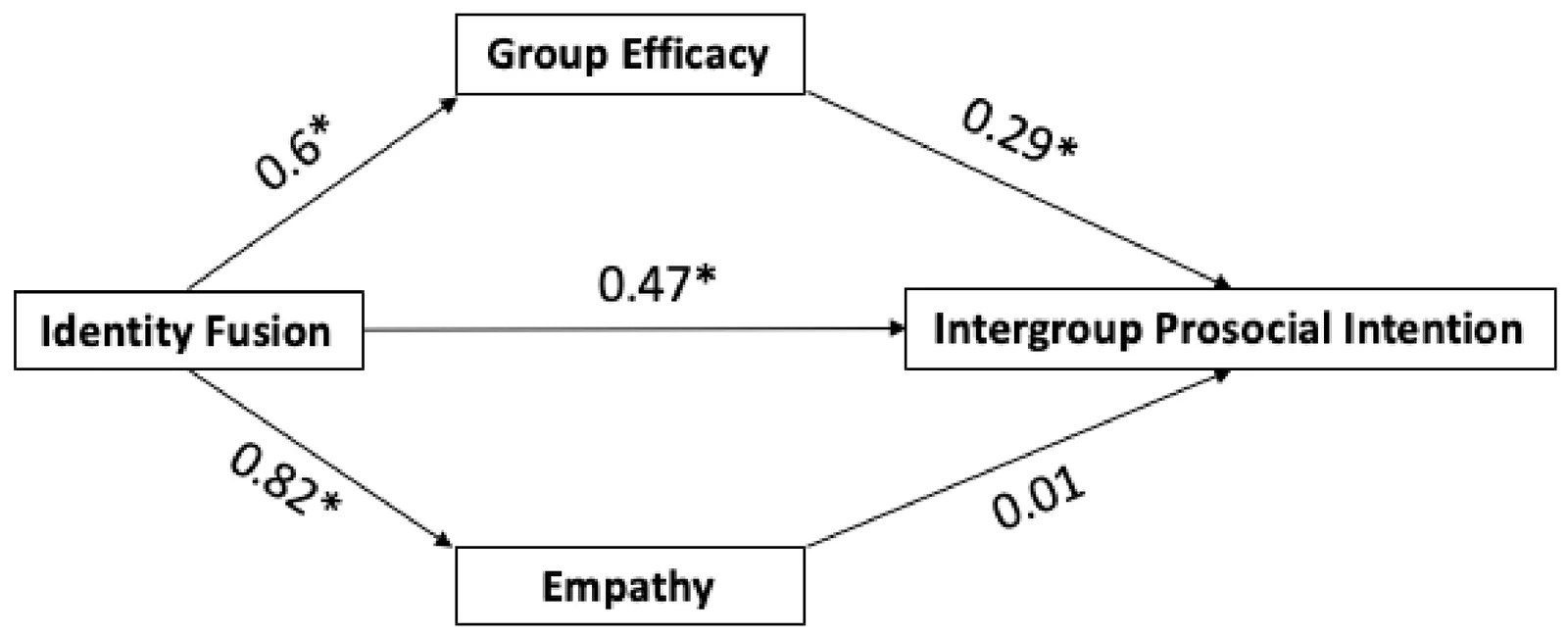
Statistical Mediation Model Path for Personal Self-Activation Group. Note: * *p* < 0.05.

In the social self-activation group, the indirect effect of group efficacy between identity fusion and intergroup prosocial intention was significant, *95% CI* [0.01, 0.37], whereas the indirect effect of empathy was not significant, *95% CI* [−0.42, 0.28]. The direct effect of identity fusion on intergroup prosocial intention was significant; thus, only group efficacy showed partial mediation, *95% CI* [0.11, 0.73] (see Table 5 and Figure 5). As shown in Table 6 and Figure 6, group efficacy again showed a significant indirect effect, *95% CI* [0.05, 0.35], whereas empathy did not, *95% CI* [−0.15, 0.30]. The direct effect of identity fusion remained significant, *95% CI* [0.24, 0.70], indicating partial mediation via group efficacy.

### 3.5. Study 2 Discussion

Study 2 provided experimental support for H4. Participants in both the social self-activation condition and the personal self-activation condition reported higher intergroup prosocial intention than those in the control condition, suggesting that activating self-representations related to identity fusion can strengthen prosocial tendencies toward outgroup members in this low-conflict regional context. The difference between the social self and personal self-activation conditions was not statistically significant, indicating that the present data do not provide evidence that one form of activation is stronger than the other. Thus, RQ1 was answered in a non-directional way: both activation conditions increased intergroup prosocial intention relative to the control condition, but they did not differ significantly from each other. Additional process analyses suggested that the group efficacy pathway was more robust than empathy in the experimental context, as the indirect effect through empathy was not significant. These results complement Study 1 by suggesting that the positive intergroup implications of identity fusion may be especially supported by perceived group efficacy, whereas the empathy pathway may be more context- or measurement-dependent. Also, given the small sample size within each condition, these mediation results should be interpreted cautiously and are discussed further in the General Discussion.

## 4. General Discussion

Taken together, the findings provide support for the central prediction of the present research. Hypothesis 1, which proposed a positive association between identity fusion and intergroup prosocial intention, was supported. The hypotheses concerning the indirect roles of group efficacy and empathy received partial support. Group efficacy showed relatively more consistent evidence across the two studies, whereas empathy showed a significant indirect association in Study 1 but was not significant in the exploratory mediation analyses in Study 2. The experimental findings in Study 2 also supported the prediction that activating identity-related self-representations would increase intergroup prosocial intention relative to the control condition, although the two active conditions did not differ significantly from each other. These findings suggest that identity fusion may contribute to intergroup prosocial intention, but that its psychological pathways and boundary conditions require further investigation.

Across two studies, we found preliminary and complementary evidence that identity fusion is positively associated with intergroup prosocial behavior and that self-activation tasks related to identity fusion were associated with differences in intergroup prosocial intention, compatible with the Fusion–Secure Base Hypothesis regarding the possibility that fusion may foster outgroup-oriented tendencies in relatively low-threat contexts. In such contexts, identity fusion may cultivate positive attitudes toward outgroups and may have implications for understanding positive intergroup tendencies. This aligns with prior findings that stronger fusion with one’s group is linked to greater prosociality, thereby contributing to social harmony (Drury et al., 2009; Vázquez et al., 2023). During the COVID-19 pandemic, highly fused individuals were more likely to encourage others to receive vaccinations (Paredes et al., 2021), and identity fusion more strongly buffered negative emotions and panic (e.g., concerns about resource shortages and infection) during uncertain crisis periods (Seih et al., 2021). The present research provides partial evidence for the “bright side” of identity fusion, suggesting that identity fusion, which has often been studied in relation to extreme pro-group behavior, may also be linked to greater willingness to engage with outgroup members in relatively low-threat contexts consistent with the Fusion-Secure Base Hypothesis. However, the present study does not provide a direct test of the secure-base mechanism because perceived threat, perceived safety, and ingroup-as-secure-base perceptions were not directly measured. Future research should directly measure or manipulate perceived intergroup threat and secure-base perceptions to examine whether fused individuals become more willing to engage with outgroup members because their ingroup provides a sense of safety and support.

The Fusion–Secure Base Hypothesis is rooted in attachment research. Whereas attachment figures traditionally refer to parents or romantic partners, this framework has increasingly been extended to broader social groups. As individuals age, starting in adolescence, groups increasingly serve as attachment figures (Delgado et al., 2022). From an evolutionary perspective, the security provided by groups is as crucial as that provided by caregivers for infants (Smith et al., 1999). As societies develop, such secure bases extend into everyday life. Accordingly, the Fusion–Secure Base Hypothesis suggests that trust in the ingroup provides support and protection (as a secure base), reducing intergroup risk. Through trust in the ingroup, fusionists may become able to trust others (including outgroup members) and pursue mutually beneficial social opportunities (Klein & Bastian, 2023).

We examined potential process mechanisms from competence–agency and affect–care perspectives. Study 1 showed significant cross-sectional indirect associations through both group efficacy and empathy. However, the Study 2 mediation findings were more limited. In the exploratory condition-specific analyses, the indirect effect through group efficacy was significant in the personal self-activation condition, and the corresponding indirect effect in the social self-activation condition was also significant and positive. The empathy pathway was not significant in either activation condition. Thus, although group efficacy showed relatively more consistent preliminary evidence than empathy, this conclusion should be interpreted cautiously.

Several features of the present design may help explain the weaker evidence for the empathy pathway. First, empathy was measured using the Interpersonal Reactivity Index, which captures general dispositional empathy rather than empathy specifically directed toward Northerners. In intergroup contexts, empathic responses are shaped by group boundaries, and people may show reduced empathy or even counter-empathic responses toward outgroup members (Cikara et al., 2014). Second, Study 2 activated personal self and social self-representations related to identity fusion, but it did not directly manipulate either group efficacy or empathy. The activation tasks may therefore have been more closely aligned with identity-related agency and group capacity than with empathic concern for outgroup members. Third, the outcome measure involved hypothetical donation scenarios, which may not strongly depend on empathic concern, especially when the recipients are presented as outgroup members rather than as personally vivid targets. Future research should use outgroup-specific empathy measures, directly manipulate empathy or empathic concern, and include helping scenarios or behavioral tasks that more clearly vary the salience of outgroup suffering and need.

Recent work on solidarity-based action further suggests that empathy in intergroup contexts may depend on how the target group’s situation is morally interpreted. For example, Borinca et al. (2026) showed that exposure to civilian suffering can increase empathy, perceived injustice, and solidarity-based action intentions, with effects varying across target groups and conflict contexts. Although their work focused on military conflicts rather than regional relations within China, it is theoretically relevant because it suggests that empathy is not only a stable individual disposition, but can also be shaped by the salience of group-based suffering, perceived injustice, and the identity of the target group. In the present studies, the helping scenarios involved outgroup members in need, but we did not directly manipulate the salience of suffering, perceived injustice, or the moral interpretation of Northerners’ situation. Future research should therefore compare general empathy, outgroup-directed empathic concern, perceived injustice, and anticipated quality of cross-group interaction as possible mechanisms linking identity fusion to intergroup prosocial intention.

In Study 2, activating social self and personal self-representations yielded a significant indirect effect via group efficacy but not via empathy, suggesting that under our priming and measurement framework, the empathy pathway may be less stable than the efficacy pathway. Importantly, Study 2 activated self-representations related to identity fusion (personal self/social self) rather than directly manipulating empathy. Thus, whether empathy functions as a stable mechanism warrants further investigation using more direct emotion/empathy manipulations and measures more closely tied to outgroup contexts. Because the social self-activation materials (Broad and Narrow Sichuan) and the accompanying imagination/writing tasks were directly related to the participants’ group, social self-activation may have elicited group efficacy to some extent but may not have further elevated empathy. Likewise, the personal self-activation materials focused on self-knowledge and did not involve deeper understanding of others’ emotions, which may also have made empathy difficult to enhance.

Furthermore, given that empathy is motivational, people may avoid empathy when it induces negative experiences or entails material costs; because intergroup prosociality in this research was expressed via donation scenarios, participants may have been motivated to avoid empathy (Zaki, 2014). This result may also reflect the small sample size and limited power of the Study 2 mediation analyses. Future studies should improve experimental designs by activating and controlling additional variables to test intergroup prosociality more directly in the affective domain and increase the sample size of the experiment to test empathy.

By experimentally inducing social self and personal self-representations, we found that both forms of activation increased intergroup prosocial tendencies relative to the control group. Social Identity Theory posits that interpersonal interactions fall along an interpersonal–intergroup continuum and that when the social self is salient, it can override the personal self and drive group-relevant behavior (Tajfel & Turner, 2004). Thus, intergroup prosocial behavior may be activated by social norms or by fusionists’ social self. Our pattern differs somewhat from Swann et al. (2014), where personal self-activation was more effective among Spanish participants with more individualistic value orientations. The North–South Chinese context provides a culturally meaningful setting for examining identity fusion and intergroup prosocial intention, but the cultural interpretation of the present findings should remain cautious. Rice theory (Talhelm et al., 2014) suggests that Southern and Northern China may differ in interdependence, holistic thinking, and related cultural orientations. However, these constructs were not measured in the present studies. Therefore, we cannot conclude that Southern Chinese participants were more interdependent, more holistic in thinking, or more responsive to social self-activation for cultural reasons. Moreover, the difference between the social self and personal self-activation conditions was not statistically significant. Thus, rice theory should be understood as providing contextual background for the regional boundary rather than as an explanation directly supported by the present data.

The practical implications of these findings are necessarily tentative. The present studies did not test prejudice reduction, durable behavioral change, or actual helping. Therefore, the findings should not be interpreted as evidence that identity fusion-based interventions can directly promote real-world intergroup cooperation. A more cautious implication is that brief self-reflection tasks related to personal or social identity may influence short-term prosocial intentions under controlled conditions. Future research should test whether such effects generalize to actual intergroup behavior and longer-term changes in intergroup attitudes.

## 5. Theoretical Implications

Studies 1 and 2 provide preliminary evidence for the applicability of the Fusion–Secure Base Hypothesis to intergroup prosocial behavior and highlight the potentially constructive role of identity fusion in intergroup relations. Moreover, using China’s North–South cultural divide as a real-world intergroup boundary offers a contextual testing window. Building on group efficacy and empathy, we developed and examined a dual-path model from intrapersonal and interpersonal perspectives, providing a new analytic lens on the internal processes underlying intergroup prosocial behavior and extending the potential theoretical value of the dual-path framework to other possible psychological correlates.

## 6. Limitations and Future Directions

Several limitations warrant attention.

First, the measurement of intergroup prosocial intention should be interpreted with caution. Although the prosocial scenarios explicitly instructed participants that the recipients were Northern Chinese residents and therefore members of a regional outgroup, all scenarios involved recipients experiencing some form of hardship, such as poverty, educational disadvantage, or disaster-related need. Thus, participants’ responses may have reflected both intergroup prosocial intention and more general humanitarian or moral concerns toward people in need. Future research could employ more neutral helping scenarios involving Northern Chinese outgroup members to better distinguish intergroup prosocial motivation from general need-based helping.

Second, the sample characteristics may limit the generalizability of the findings. Participants were limited to young adults aged 18–35, and future work should examine whether the present findings extend to children, adolescents, and older adults. In addition, Study 2 used a selected subsample from the Study 1 participant pool. Although this strategy was intended to reduce ceiling effects, it may have restricted the generalizability of the findings, introduced regression-to-the-mean effects, and created potential carry-over effects. These concerns cannot be fully ruled out, although the two studies were separated by approximately four months and participants were randomly assigned to experimental conditions. Future studies should replicate the experiment with an independent and more representative sample.

Third, several features of the research design constrain causal interpretation and statistical inference. Study 1 was cross-sectional and therefore cannot establish causal relationships; longitudinal designs are needed to examine the temporal ordering among identity fusion, group efficacy, empathy, and intergroup prosocial intention. Although Study 1 analyses controlled for basic demographic variables, we did not measure several factors that may plausibly influence both identity fusion and intergroup prosocial intention. These include perceived intergroup threat, perceived similarity between Southerners and Northerners, prior contact with Northerners, strength of regional identification, socioeconomic background, and political or cultural attitudes toward Northern China. Given that these variables were not assessed, we cannot rule out the possibility that they partly account for the observed associations. Future research should include these variables to clarify whether identity fusion predicts intergroup prosocial intention above and beyond broader intergroup attitudes, contact experiences, perceived threat, and regional identity strength. Moreover, the sample size of Study 2 was limited. Although the study was designed to detect relatively large condition effects, this sample size provides limited power for mediation analyses and for directly comparing the two active activation conditions. Therefore, the mediation analyses in Study 2 should be interpreted as exploratory, and the non-significant difference between the social self and personal self-activation conditions should not be taken as evidence of equivalence. Future studies should replicate these findings with larger samples and preregistered analytic models.

Relatedly, although the present research was guided by the Fusion–Secure Base Hypothesis, we did not directly measure perceived intergroup threat, perceived safety, security, or ingroup-as-secure-base perceptions. Therefore, the present findings cannot determine whether identity fusion was associated with intergroup prosocial intention because the ingroup functioned as a secure base. Future studies should include direct measures or manipulations of perceived threat and secure-base perceptions to test this mechanism more rigorously.

Fourth, the experimental manipulation in Study 2 should be interpreted with caution. Although the social self condition explicitly framed participants’ hometown as part of their Southern Chinese regional identity, the materials used in the social self and personal self conditions were not fully matched in emotional tone, cultural content, vividness, positivity, or group relevance. Thus, although the manipulation was intended to activate Southern regional identity-related self-representations, alternative explanations related to positive affect, cultural salience, hometown pride, or demand characteristics cannot be fully ruled out. Future research should use more closely matched materials and include manipulation checks for affect, arousal, perceived group salience, and identity fusion. In addition, the present study manipulated components of identity fusion, namely the personal self and social self, but did not experimentally manipulate group efficacy and empathy. Future studies may manipulate these variables directly to examine interactions among additional mechanisms.

Finally, the cultural and identity context of the present research should be examined more broadly in future work. Identity fusion remains a relatively new construct in China; given China’s complex social groups, future research could examine diverse populations across ethnicity, occupation, and sexual orientation. Although this study focused on the regional boundary between Southern and Northern Chinese, participants may also have perceived Northerners as part of a broader shared Chinese identity. Because superordinate national identity was not directly measured, we cannot determine its potential influence on the present findings. Although the manipulation check in Study 2 indicated changes in identity fusion following the activation task, it cannot rule out the possibility that broader Chinese national identity also shaped participants’ prosocial intentions. In addition, although gender was included as a covariate in the mediation analyses, the small sample size in Study 2 limits our ability to fully rule out gender-related explanations. Future research should assess both regional and superordinate national identities, as well as potential gender-related differences, to clarify how different levels of group identity shape intergroup prosocial intention.

## 7. Conclusions

Highly fused individuals tend to report higher intergroup prosocial intentions. This tendency may operate through two psychological pathways: a competence–agency pathway via group efficacy and an affect–care pathway via empathy. Across the two studies, the group efficacy pathway received relatively more consistent support, whereas evidence for the empathy pathway was more limited and warrants further examination. Regarding the self-representational components of identity fusion, activating either the social self or the personal self increased intergroup prosocial tendencies relative to the control condition. Although the social self condition showed a higher mean than the personal self condition, this difference did not reach significance and should be examined further using more tightly matched materials and larger samples.

## Figures and Tables

**Table 1:** Correlations Across Key Variables in Study 1.

	*M ± SD*	*1*	*2*	*3*	*4*
1. Identity fusion	37.24 ± 8.97	1			
2. Group efficacy	21.31 ± 5.48	0.75 ***	1		
3. Empathy	78.98 ± 10.13	0.13 ***	0.11 ***	1	
4. Intergroup prosocial behavior	23.08 ± 4.57	0.40 ***	0.37 ***	0.38 ***	1

Note: *** *p* < 0.001.

**Table 2:** Analysis of Mediating Effects in Study 1.

Mediation Pathway	*Effect*	*BootSE*	*Efficiency Ratio*	*95% CI*	
				*BootLLCI*	*BootULCI*
X → M1 → Y	0.06	0.02	28.57%	0.02	0.10
X → M2 → Y	0.02	0.01	9.52%	0.01	0.04
Direct Effect	0.12	0.03	57.14%	0.07	0.17
Total Effect	0.21	0.02	100%	0.17	0.24

Note. Path 1: Identity fusion → group efficacy → intergroup prosocial intention. Path 2: Identity fusion → empathy → intergroup prosocial intention, below. Bootstrapped indirect, direct, and total effects are reported as unstandardized estimates.

**Table 3:** Identity Fusion on the Pre-test.

Pre-Test of Identity Fusion	*M ± SD*	*F*
Social Self-Activation Group (*N* = 24)	35.50 ± 4.16	
Personal Self-Activation Group (*N* = 24)	34.21 ± 3.96	0.73
Control Group (*N* = 24)	35.38 ± 4.12	

**Table 4:** Identity Fusion on the Post-test.

Post-Test of Identity Fusion	*M ± SD*	*F*
Social Self-Activation Group (*N* = 24)	38.21 ± 4.23	
Personal Self-Activation Group (*N* = 24)	35.67 ± 3.60	5.94 **
Control Group (*N* = 24)	34.42 ± 3.80	

Note: ** *p* < 0.01.

**Table 5:** Analysis of Mediating Effects for Social Self-Activation Group in Study 2.

Mediation Pathway	*Effect*	*BootSE*	*Efficiency Ratio*	*95% CI*	
				*BootLLCI*	*BootULCI*
X → M1 → Y	0.14	0.09	28%	0.01	0.37
X → M2 → Y	−0.06	0.18	−12%	−0.42	0.28
Direct Effect	0.42	0.15	84%	0.11	0.73
Total Effect	0.50	0.05	100%	0.40	0.60

**Table 6:** Analysis of Mediating Effects for Personal Self-Activation Group in Study 2.

Mediation Pathway	*Effect*	*BootSE*	*Efficiency Ratio*	*95% CI*	
				*BootLLCI*	*BootULCI*
X → M1 → Y	0.17	0.08	26.15%	0.05	0.35
X → M2 → Y	0.01	0.11	1.54%	−0.15	0.30
Direct effect	0.47	0.11	72.31%	0.24	0.70
Total effect	0.65	0.06	100%	0.52	0.78

## Data Availability

The data presented in this study are available from the corresponding author upon reasonable request. The data are not publicly available due to privacy and ethical restrictions related to participant confidentiality.
